# A New Computational Deconvolution Algorithm for the Analysis of Forensic DNA Mixtures with SNP Markers

**DOI:** 10.3390/genes13050884

**Published:** 2022-05-15

**Authors:** Yu Yin, Peng Zhang, Yu Xing

**Affiliations:** 1 Department of Forensic Medicine, Chongqing Medical University, #1 Yixueyuan Road, Chongqing 400016, China; yhazel@163.com (Y.Y.); zpcmu6666@163.com (P.Z.); 2 Public Security Bureau of Chongqing Nanchan District, #11 Jinshan Avenue, Nanchang District, Chongqing 408499, China

**Keywords:** forensic genetics, bioinformatics, single nucleotide polymorphism (SNP), massively parallel sequencing (MPS), Precision ID Identity Panel, DNA mixture deconvolution, K-means clustering, SAMtools

## Abstract

Single nucleotide polymorphisms (SNPs) support robust analysis on degraded DNA samples. However, the development of a systematic method to interpret the profiles derived from the mixtures is less studied, and it remains a challenge due to the bi-allelic nature of SNP markers. To improve the discriminating power of SNPs, this study explored bioinformatic strategies to analyze mixtures. Then, computer-generated mixtures were produced using real-world massively parallel sequencing (MPS) data from the single samples processed with the Precision ID Identity Panel. Moreover, the values of the frequency of major allele reads (*F_MAR_*) were calculated and applied as key parameters to deconvolve the two-person mixtures and estimate mixture ratios. Four custom R language scripts (three for autosomes and one for Y chromosome) were designed with the K-means clustering method as a core algorithm. Finally, the method was validated with real-world mixtures. The results indicated that the deconvolution accuracy for evenly balanced mixtures was 100% or close to 100%, which was the same as the deconvolution accuracy of inferring the genotypes of the major contributor of unevenly balanced mixtures. Meanwhile, the accuracy of inferring the genotypes of the minor contributor decreased as its proportion in the mixture decreased. Moreover, the estimated mixture ratio was almost equal to the actual ratio between 1:1 and 1:6. The method proposed in this study provides a new paradigm for mixture interpretation, especially for inferring contributor profiles of evenly balanced mixtures and the major contributor profile of unevenly balanced mixtures.

## 1. Introduction

A DNA mixture is known as a biological sample that originates from two or more donors and is determined after a DNA profile is generated [1]. In practice, mixed DNA stains are common evidence in criminal cases involving a victim and a perpetrator (e.g., sexual assault evidence, fingernail cuttings taken by police, etc.,) [1,2]. However, the analysis and interpretation of DNA mixtures from crime scenes remain a significant challenge due to the uncertainties in the number of donors and their relative proportions, and the challenge is further intensified by low-quality samples [3,4]. Short tandem repeat (STR) polymorphisms have been widely used as the mainstay genetic marker for forensic mixture analysis [5]. There are various software tools available to interpret mixtures with STR markers, not only providing a likelihood ratio (LR) to express the weight of evidence but also deconvolving profiles, such as EuroForMix and STRmix^TM^ [6,7,8]. However, due to allelic drop-out and drop-in, STR markers show some deficiency in analyzing degraded DNA mixtures that usually occur in routine casework [9,10,11,12]. In addition, the presence of stutter products in STR amplification is prone to be incorrectly considered as alleles of minor contributors, which further complicates the analysis of STR profiles [5]. Fortunately, single nucleotide polymorphisms (SNPs) exhibit robust analysis on degraded DNA samples, and it requires smaller target regions than STR markers [13]. In addition, SNPs are widespread in the human genome with a lower mutation rate and can provide extra genetic information attributing to parental lineage determination, biogeographical ancestry, or phenotypical traits assessment [14]. Moreover, the introduction of the massively parallel sequencing (MPS) technology makes SNPs more powerful in handling degraded or trace samples based on its exportation of detailed and quantitative sequence information, even with incomplete DNA fragments [15,16]. Further, more SNP-MPS commercial kits that have been available since 2013, are commonly used in comparison to microhaplotype, mitochondrial DNA, or indel (i.e., insertion or deletion) markers [5,17,18]. However, the bi-allelic nature limits the ability of SNP markers to interpret DNA mixtures in the forensic community [13,19]. Methods for analyzing mixtures with SNPs were also restricted to calculating LR using statistical theory in the case of known suspects, and deconvolution analysis was rarely performed using bioinformatics tools [20,21,22,23].

Given the comparison results above, to improve the discriminating power and overcome the shortcoming of SNPs, this study explored bioinformatic strategies to analyze DNA mixtures and made some new progress. First, 825 in silico two-person mixtures varying in 28 ratios were created by using six single-source binary alignment map (BAM) files generated from the Precision ID Identity Panel on the MPS platform. The Precision ID Identity Panel (formerly known as HID-Ion AmpliSeq™ Identity Panel) released by Thermo Fisher Scientific was designed to detect 90 autosomal SNPs (A-SNPs) from the K.Kidd’s 45-unlinked set [24] and the SNPforID set [25], as well as 34 upper Y-clade SNPs (Y-SNPs) [26]. This panel has been evaluated by numerous studies and considered to fully meet the requirements of individual identification and paternity testing in forensic science [27,28,29,30,31]. In addition, the average amplicon sizes of A-SNPs and Y-SNPs were 132 bp and 141 bp, respectively [32]. Then, the K-means clustering algorithm was utilized to process the values of the frequency of major allele reads (*F_MAR_*), which for each locus was calculated as the largest reads among the four bases divided by the total detected reads [33]. These values were used for deconvolving in silico mixtures and estimating mixture ratios. The *F_MAR_* of single-source DNA sequencing data should be approximately 50% (heterozygote) or 100% (homozygote and Y-SNPs), while that of two-person mixtures should follow a different rule [32,33]. Finally, this bioinformatic pipeline was evaluated through in vitro two-person mixtures analysis, and accurate results were obtained.

## 2. Materials and Methods

### 2.1. Samples, SNP Typing, and Data Description

Blood samples from four individuals (i.e., P1, P2, P3, and P4), all females except that P1 was male, were collected with their informed consent and used as single-source samples along with two other male samples, DNA 007 (Thermo Fisher Scientific, Waltham, MA, USA) and 2800 M DNA (Promega, Madison, WI, USA). Real mixtures of 2800 M and 9948 male DNA (Promega) were generated at ratios of 19:1, 9:1, 4:1, 1:1, 1:4, 1:9, and 1:19 to test the reliability of the bioinformatic pipeline in this study.

DNA was extracted by the AutoMate Express^TM^ Forensic DNA Extraction System with the PrepFiler Express^TM^ Forensic DNA Extraction Kit. The extracted DNA was quantified on the QuantStudio^TM^ 5 Real-Time PCR System using the Quantifler^®^ Trio DNA Quantification Kit (both from Thermo Fisher). For each single and mixture sample, library construction was performed on the Ion Chef^TM^ Instrument using the Precision ID Identity Panel (both from Thermo Fisher) to hold the amount of the input DNA at 1 ng. Libraries were then quantified with the Ion Library TaqMan^TM^ Quantification Kit (Thermo Fisher). Finally, the constructed libraries were templated onto the Ion Sphere Particles via the Ion Chef^TM^ Instrument and loaded onto an Ion 530^TM^ chip, which was sequenced on the HID Ion GeneStudio^TM^ S5 Prime System platform (both from Thermo Fisher) [34].

After sequencing, BAM and binary alignment index (BAI) files were obtained through processing with the Torrent Suite Server (Thermo Fisher). The BAM file is the binary version of a sequence alignment map (SAM) file, and it contains aligned reads sorted by reference location (reference genome GRCh37/hg19). Especially, the BAM file is compact and supports fast retrieval of alignments in targeted regions. Thus, using positional sorting and indexing, applications can perform stream-based processing on specific genomic regions without loading the entire file into memory [34,35]. By contrast, a SAM file is a tab-delimited text file that is slower to parse, and it consists of one header section and one alignment section. The lines in the header section start with the character “@”, and the lines in the alignment section have eleven mandatory fields. In each alignment line, SNP markers are found in three fields, i.e., RNAME (reference sequence name), POS (leftmost mapping position on the reference sequence), and SEQ (segment sequence). The BAM file and SAM file can be converted to each other with the help of SAMtools [35]. 

The sequencing data were further processed by the HID SNP Genotyper_5_2_2 plugin (Thermo Fisher) with default settings. The comma-separated value (CSV) files created by the plugin contain the following information for each of the 124 loci: position information, the number of reads for each of the four bases, total coverage of aligned reads, strand bias, genotype, *F_MAR_*, genotype quality, etc. Among them, strand bias was measured as the ratio of forward strand coverage to total coverage. The value outside the range of 30% to 70% indicates strand imbalance, and the value of 50% indicates perfect strand balance. In addition, the *F_MAR_* values for homozygotes (Hom) were above 95%, while *F_MAR_* values for heterozygotes (Het) were between 35% and 65% [32]. All CSV files revealed that most of the samples showed no calls (NN) for rs2269355 and rs1523537 because the total coverage of aligned reads was less than the threshold (minimum of 20 reads for A-SNPs and 10 reads for Y-SNPs). Therefore, these two loci (rs2269355 and rs1523537) were excluded from this study together with rs7520386, which was genotyped inconsistently in the related MPS studies [27,28,36]. As a result, 121 loci (87 A-SNPs and 34 Y-SNPs) remained for each sample. Figures of the overall performance of the six single-source samples in terms of coverage, strand bias, and *F_MAR_* are shown in Supplementary File S1.

### 2.2. Theoretical F_MAR_ Values at Different Mixture Ratios

There are only three kinds of genotypes for each locus of the bi-allelic A-SNPs, i.e., a heterozygote and two different homozygotes. Therefore, when two random individuals are mixed at the ratio of 1:1, only three theoretical *F_MAR_* values (*TF*) are generated, i.e., 100%, 75%, and 50%, as shown in Table 1. Assuming that the alleles of a locus of A-SNPs are A and G, all possible combinations at the ratio of 1:1 were listed in Table 1. Thus, the following three points can be summarized in the 1:1 two-person mixture: (a) If *TF* is equal to 100%, then the locus is mixed by the identical homozygotes; (b) if *TF* is equal to 75%, then the locus is mixed by a heterozygote and a homozygote, where the homozygote contains the allele with the maximum amount in this site; (c) if *TF* is equal to 50%, then the locus is mixed by different homozygotes or identical heterozygotes. 

**Figure 1:**
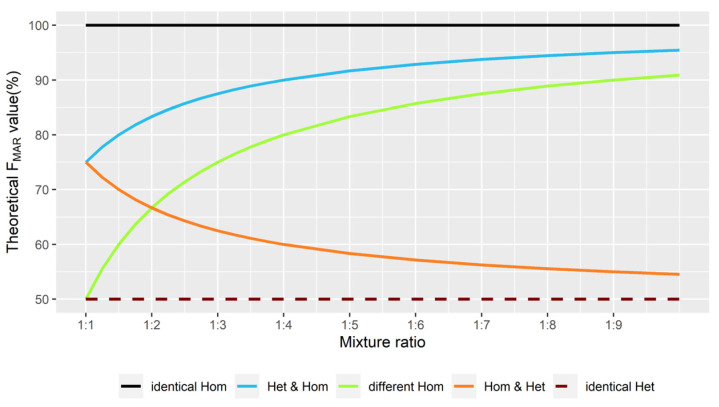
Illustration of the mixture ratio (*x*-axis) against the theoretical *F_MAR_* values (*y*-axis). The five lines in the graph are interpreted as follows. First, the black solid line at the top indicates *TF1*, which always equals 100%. Second, the blue line indicates *TF2*, which becomes higher and is infinitely close to 100%, as the proportion of the major contributor increases. For example, *TF2* equals 95% and 97.5% at the ratios of 1:9 and 1:19, respectively. Third, the green line indicates *TF3*, which becomes higher and is infinitely close to *TF2*. *TF3* equals 90% and 95% at the ratios of 1:9 and 1:19, respectively. Fourth, the red solid line indicates *TF4* which becomes lower and is infinitely close to 50%. *TF4* equals *TF2* at the ratio of 1:1, and it equals *TF3* at the ratio of 1:2. Fifth, the brown dashed line at the bottom indicates *TF5*, which always equals 50%.

According to the change rule in the number of the two alleles at different mixture ratios, Equation (1) regarding *TF* was derived. By transforming Equations (1) and (2), the mixture ratio was calculated.

(1) $$\mathrm{TF}=0.5\;\times \;\frac{k_{\mathrm{co}}}{n+1}+0.5,\;n\;\geq {\;1,\;k}_{\mathrm{co}}\;\in \;\left\{0,\;1,\;n-1,\;n,\;n+1\right\}$$

where *TF* denotes the theoretical *F_MAR_* values of A-SNP. The variable *n* indicates the mixture ratio 1:*n*, and it can be any number greater than or equal to 1, including decimals. Three of the coefficient “k_co_” of Equation (1) are determined by the variable *n*. When the mixture ratio is fixed (i.e., *n* is unique), the relationship between k_co_, *TF*, and the genotype combinations is shown in Table 2. If *n* = 1 (at the ratio of 1:1), then from Equation (1), *TF1* = 100%, *TF2* = *TF4* = 75%, *TF3* = *TF5* = 50% (complete details in Figure 1).

(2) $$n=\frac{\mathrm{TF}2\;-\;0.5}{1\;-\;\mathrm{TF}2}=\frac{\mathrm{TF}3}{1\;-\;\mathrm{TF}3}=\frac{1\;-\;\mathrm{TF}4}{\mathrm{TF}4\;-\;0.5}$$

where *n* is calculated from Equation (2) by taking different values of the coefficient k_co_ into Equation (1).

### 2.3. Simulations with In Silico Mixtures 

If the actual *F_MAR_* values of the two-person mixtures are the same as the theoretical values, then deconvolution will be easy. However, in realistic single-source sequencing data, due to the background signals from non-alleles and heterozygote allelic imbalance caused by stochastic effects in PCR, *F_MAR_* values are below 100% in some homozygous loci and above 50% in most heterozygous loci [27,28,31,37], i.e., there is a gap between the theoretical and actual values of *F_MAR_* for a single sample. Therefore, *TF* of the mixture is also different from the actual one, although there is a clear correlation between them [33]. In order to study *F_MAR_* more easily and comprehensively, and to use it for deconvolution analysis, sufficient mixtures with various ratios were simulated on the computer.

Two single samples were taken from the six samples at a time, producing in silico mixtures with 15 distinct combinations at the ratio of 1:1 and 30 distinct permutations at each of the other ratios. A total of 825 independent mixtures were created at 28 varying ratios between 1:1 and 1:19 (details in Supplementary File S2). Among them, the interval of the ratios between 1:1 and 1:4 was 0.25 (e.g., 1:1.25, 1:1.50, 1:1.75) and the remaining intervals were 1 (e.g., 1:4, 1:5, 1:6). The reason for designing a smaller value for the ratio interval between 1:1 and 1:4 was that the *TF* lines in Figure 1 crossed in this range. This design was used to carefully observe whether the deconvolution accuracy was affected when the *TF* values of different genotype combinations were the same. On the other hand, a study by Guo et al. showed that the panel could not obtain the full profile from minor contributor at the ratio of 1:19 with 1 ng of total DNA input [27]. Therefore, in silico mixtures with more extreme proportions were not created.

The variations in coverage among samples and loci due to the pooling of libraries and PCR should be taken into account during simulation [38]. Therefore, the strategy was to use P1, which had the lowest mean locus coverage and standard deviation (A-SNPs: 675× ± 328×, Y-SNPs: 351× ± 173×) among the six samples, as a template and create in silico mixtures on the Linux operating system through a series of commands, shells, and Python scripts (version 3.6.6). Especially, to be more like the real-world products, the coverage of each locus in all computer-generated mixtures was the same as that of P1. More importantly, the way simulated the coverage variations of each locus, locus strand, and heterozygous allele, and even simulated the noise level of each locus. The simulation strategy was completed in three steps, i.e., splitting, extracting, and merging of BAM files (see Supplementary File S3).

#### 2.3.1. Splitting BAM Files

The target sequences containing SNP sites were heavily amplified during library construction, but a small number of sequences without SNP sites were also involved in the BAM file. To find the amplicons for each locus and save them as a separate file, this study used SAMtools version 1.7 to view and convert the BAM format. Meanwhile, a pipeline consisting of a series of Linux commands was used to process the sequencing data from six single-source samples [35,39,40]. After this, 121 subfiles for each male sample and 87 subfiles for each female sample were generated from each BAM file, and each subfile contained all target sequences regarding a locus. 

#### 2.3.2. Extracting and Merging

The Linux command “shuf” was used to randomly extract sequencing reads from 121 subfiles according to the proportion of the coverage per locus of P1, and the software package SAMtools was applied to merge different samples [35,39,40]. For example, the reads of rs490413 and rs4847034 in P1 were stored in subfile_first.sam and subfile_second.sam, and their coverage was 554× and 471×, respectively. First, 277 sequencing reads (half of 554) from subfile_first.sam and 236 sequencing reads (approximately half of 471) from subfile_second.sam were randomly extracted, and they were stored in half_first.sam and half_second.sam, respectively. Then, the rest of the subfiles were processed in the same way. After a series of commands processing, the 121 newly generated files were combined into a new BAM file called P1_half.bam. Therefore, the coverage of each locus in P1_half.bam was half less than that of P1.bam. In addition, P2_half.bam, P3_half.bam, P4_half.bam, 007_half.bam, and 2800 M_half.bam were produced in the same way, and the coverage per locus of these files was the same as that of P1_half.bam. Finally, the in silico mixture of P1 and P2 at the ratio of 1:1 was created by merging P1_half.bam and P2_half.bam. Following the above workflow, all the in silico mixtures were generated.

#### 2.3.3. Generating CSV Files and Evaluating Simulations 

All BAM files were processed with the “samtools mpileup” command, producing files in “pileup” textual format. These files contain information such as the depth of coverage and bases at each position from aligned reads [41]. Then, a custom Python script called genoinfo.py was written to generate CSV files by manipulating these files (see Supplementary File S4). This script worked similarly to the HID SNP Genotyper_5_2_2 plugin, which mainly used the NumPy (version 1.19.2) and Pandas (version 1.1.3) libraries.

Finally, each process of the simulation was evaluated with respect to the available sequence reads of subfiles, the *F_MAR_* values of proportion files, and the strand bias of in silico mixtures. In the process of splitting BAM files, it was checked whether each subfile contained all sequence reads for each locus or contained some useless sequence reads. During the extracting process, the *F_MAR_* values of proportion files (e.g., P1_half.csv or P2_half.csv) were calculated to evaluate allele read frequency balance of homozygotes and heterozygotes. As well as the performance of strand bias of in silico mixtures was used to assess locus strand balance in the merging process.

### 2.4. Clustering F_MAR_ with K-Means Method 

Since the *F_MAR_* values of diploid and haploid in the mixture were significantly different, A-SNPs and Y-SNPs were analyzed separately. 

#### 2.4.1. A-SNP

**Figure 2:**
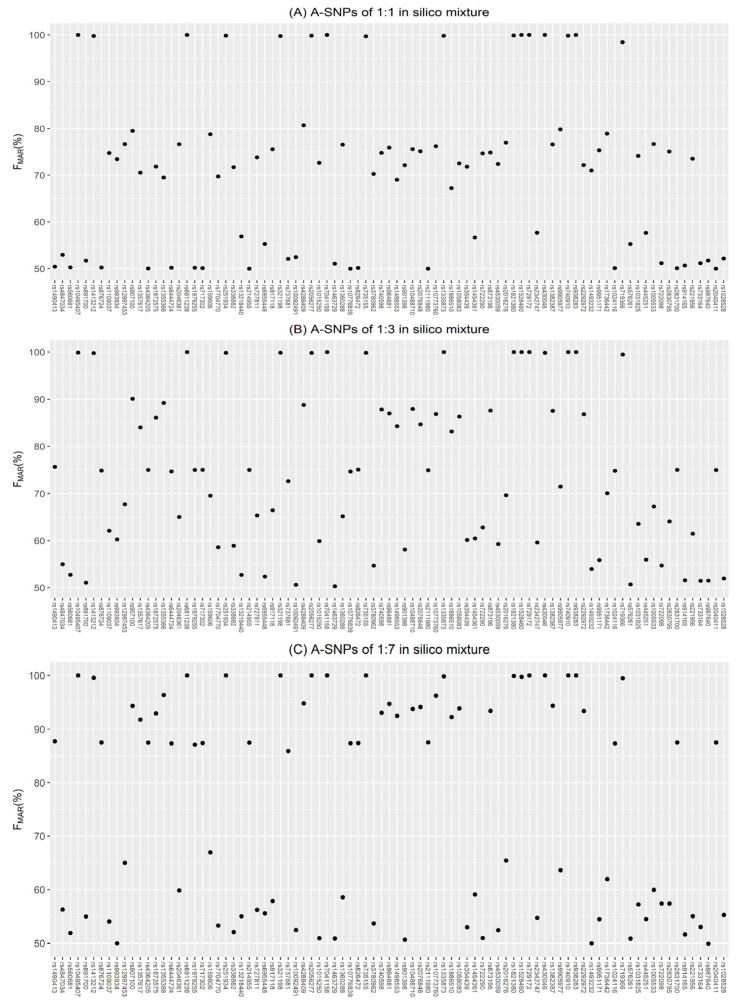
Examples of *F_MAR_* values for three in silico mixtures. (**A**) The points were mainly concentrated in three regions: the first cluster was close to 100% of the *y*-axis, the second cluster was between 70% and 80%, and the third cluster was between 50% and 60%. (**B**) The points were scattered in the range of 50% to 100% with no obvious clustering trend. (**C**) There were almost no points between 70% and 80%, and the points were clustered above 80% and below 70%, which could be considered as clustering into two categories.

**Figure 3:**
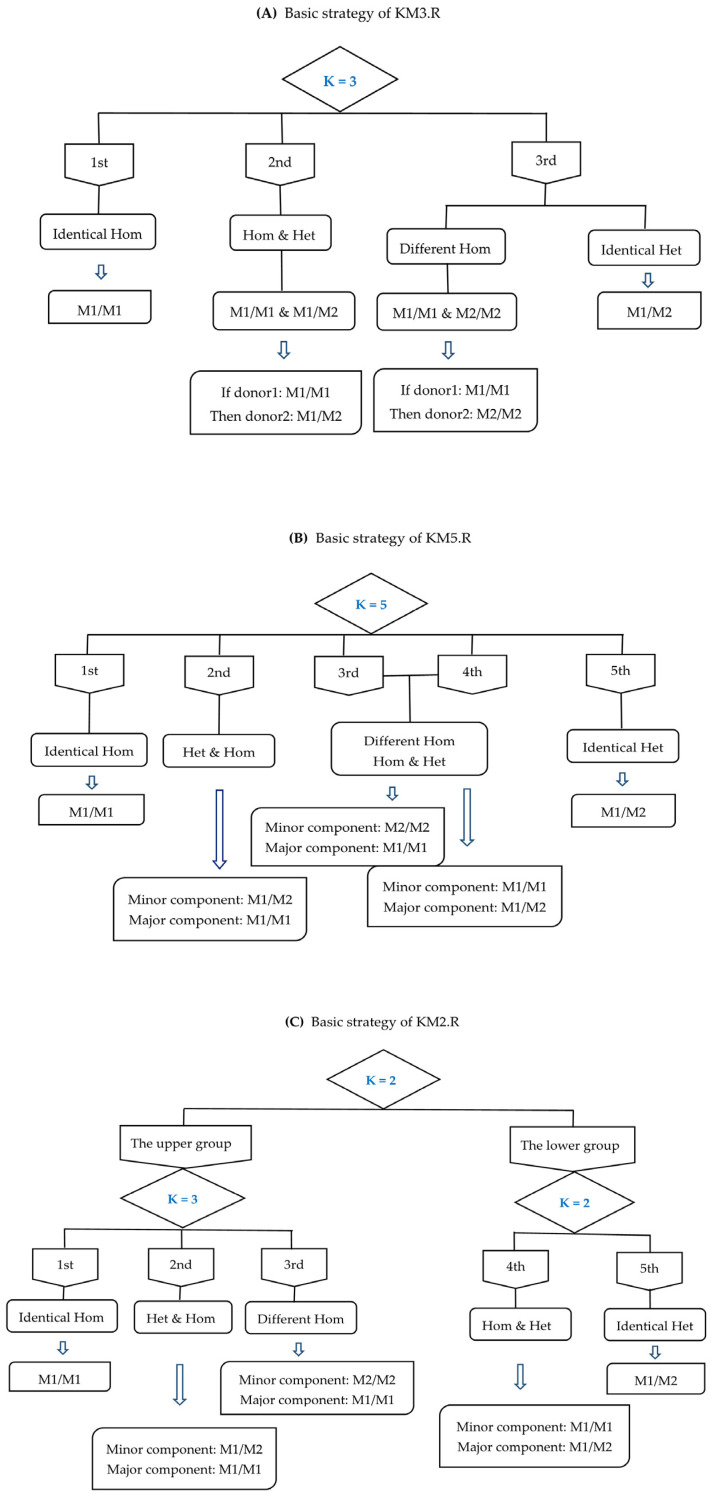
Flow charts of the basic strategies utilized for deconvolution. (**A**–**C**) represent the KM3.R, KM5.R, and KM2.R scripts, respectively. Based on the strategies, the scripts were further optimized to effectively deconvolve the incorrectly clustered loci. The value of K indicates the number of clusters needed for the dataset consisting of the *F_MAR_* values of different loci. In the KM2.R script, the *F_MAR_* values were preliminarily clustered into two groups labeled “the upper group” and “the lower group”. Then, the upper group was clustered into three categories labeled 1st, 2nd, and 3rd, and the lower group was clustered into two categories labeled 4th and 5th. Note that the meanings of M1 and M2 in the figures are the same as those in Table 1.

Scatterplots were drawn for the *F_MAR_* values of the in silico mixtures to observe the clustering. As the proportion of the major contributor increased, the scatterplot was characterized by three scenarios in turn, as shown in Figure 2A–C. The mixtures of these three scenarios were evenly balanced, mildly and moderately imbalanced, and severely imbalanced, and they were handled by the corresponding three custom scripts called KM3.R, KM5.R, and KM2.R, respectively. These scripts applied the kmeans() function of the R programming language (version 4.0.5) to perform K-means clustering analysis on *F_MAR_* values. The basic strategies of the three scripts were illustrated in Figure 3, where the value of K indicated the number of clusters needed for the dataset consisting of the *F_MAR_* values of different loci.

When a scatterplot of the *F_MAR_* values was similar to Figure 2A with all points mainly concentrated in three regions, the KM3.R script was executed. All A-SNPs were classified into three categories, each with a central value (e.g., 99.68%, 74.66%, 53.09%) and labeled as 1st, 2nd, and 3rd in order according to the size of the central values. Subsequently, deconvolution was performed as follows: the locus in the 1st category was mixed by the identical homozygotes, the locus in the 2nd category was mixed by a homozygote and a heterozygote, and the locus in the 3rd category was mixed by the different homozygotes or the identical heterozygotes. 

Although there were no apparent five clusters in Figure 2B, the *F_MAR_* values of the five different genotype combinations still had inherent differences. Accordingly, when all points were scattered in the range of 50% to 100% with no obvious clustering trend, the corresponding processing script KM5.R was applied to cluster all A-SNPs into five categories for deconvolution. According to the size of the central value of each category, they were labeled as 1st, 2nd, 3rd, 4th, and 5th in order. Among them, 1st, 2nd, and 5th categories corresponded to *TF1*, *TF2*, and *TF5* in Figure 1, respectively. The central value of 3rd category was always greater than that of 4th category, while the lines of *TF3* and *TF4* crossed at the ratio of 1:2. Therefore, 3rd category and 4th category corresponded to *TF3* and *TF4* only when the mixture ratio was greater than 1:2. With this in mind, the deconvolution strategies for 3rd and 4th categories were the same.

When a scatterplot of the *F_MAR_* values was similar to Figure 2C with all points mainly concentrated in two regions, the KM2.R script was executed. First, all A-SNPs were classified into “the upper group” that had the higher central value, and “the lower group” that had the lower central value. Then, the loci of the upper group were clustered into three categories labeled 1st, 2nd, and 3rd, and the loci of the lower group were clustered into two categories labeled 4th and 5th. Severely imbalanced in silico mixtures exhibited this type of scatterplot, so that 1st category to 5th category of KM2.R corresponded to *TF1* to *TF5* in Figure 1, respectively.

For all in silico mixtures, the genotypes of the major components were first inferred under the assumption that the genotypes of the minor components had been known, and then the opposite situation was handled. Finally, the accuracy of the inferred genotypes was detected. Additionally, KM3.R, KM5.R, and KM2.R all executed an identical command, which substituted the central value of the 2nd category of each mixture as *TF2* into Equation (2) to calculate the proportion “*n*”. Thus, running each script once resulted in both the estimated mixture ratio and the inferred genotypes. The estimated mixture ratios were then analyzed by linear regression. 

#### 2.4.2. Y-SNP

If two males have the same biogeographic origin, then the deconvolution of the 34 Y-SNPs is meaningless. However, Y-SNPs can be easily deconvolved if the donors have different genotypes. In this study, there were seven different Y-SNPs between P1 and 007, 007 and 2800 M, as well as six different Y-SNPs between P1 and 2800 M. According to the rules of combination and permutation, there were three in silico mixtures at the ratio of 1:1, and six in silico mixtures at each of the other ratios. A custom script Y-KM2.R was designed to analyze the *F_MAR_* values of 34 Y-SNPs by using the K-means clustering method, and the number of clusters was set to 2 (see Supplementary File S1). For the loci of Y-SNPs, the cluster with higher (lower) central values implied that the two samples had the same (different) genotypes.

### 2.5. Validation with In Vitro Mixtures

For the seven in vitro mixtures, 2800 M and 9948 were assumed to be a known victim and an unknown perpetrator, respectively. Since the scatterplot for the 1:1 mixture was similar to Figure 2A, the scatterplots for the 2800 M and 9948 mixtures in the 1:4 and 4:1 ratios were similar to Figure 2B, and the scatterplots for the remaining four mixtures were similar to Figure 2C, the corresponding scripts for analyzing the A-SNPs were chosen to run. In addition, the script Y-KM2.R was used to infer Y-SNPs of 9948. Finally, the accuracy of the inferred genotypes and mixture ratios was calculated.

## 3. Results and Discussion

### 3.1. Evaluation of Simulations

A total of 624 subfiles were generated during the operation of splitting the BAM files. There was no loss of sequencing reads containing SNPs sites. However, except for 207 subfiles, there was a small number of reads without SNPs sites in the other subfiles, mainly due to false positive deletions of loci bases from amplicons. The 25th percentile, median, and 75th percentile of the percentage of invalid reads in each subfile were 0%, 0.099%, and 0.362%, respectively, with the maximum value of 9.485% located at rs737681. The sequence context surrounding rs737681 is ACC[T/C]TCA. The allele C was incorrectly detected as a false positive deletion, probably because it is located in a homopolymer region.

In the study, 55 proportion files were generated for each sample during the extracting process to form 28 mixture ratios. The variations of allele read frequency balance of heterozygotes and homozygotes in proportion files from the six samples were displayed in Supplementary File S1. As the number of extracted reads decreased, the outliers increased because of low coverage and allelic imbalance. For example, the *F_MAR_* value of the heterozygote at rs2342747 exceeded 95% in eight proportion files of three samples, i.e., P2, P3, and 2800 M. This was because all the six samples had coverage of below 200× at this locus. Especially, P1 had coverage of 52×. 

**Figure 4:**
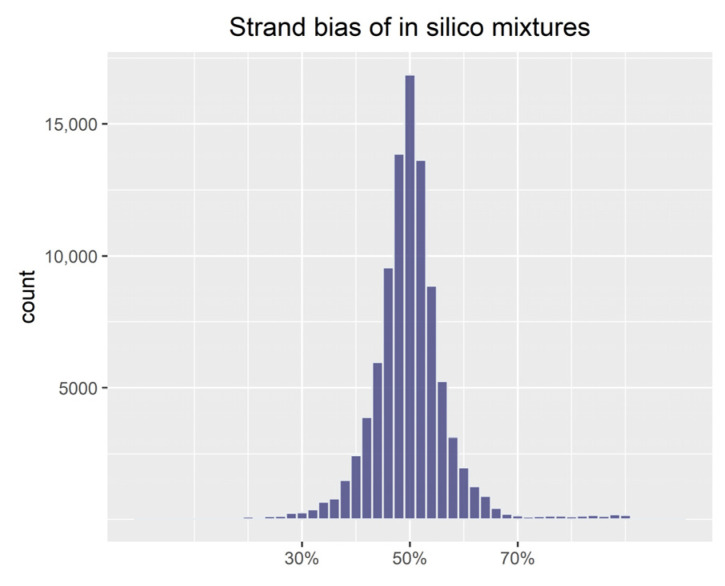
Performance of strand bias for all in silico mixtures. The values were concentrated at 50%, with 1.06% of all values outside the thresholds of 30% and 70%.

As shown in Figure 4, the strand bias values for in silico mixtures were concentrated at 50%, and 1.06% of the values were outside the thresholds of 30% and 70%. The strand bias values from in silico mixtures (median 49.84%) and the original samples (median 49.77%) were compared by using the wilcox.test() function (Wilcoxon rank-sum test) in R. The comparison result indicated that there was no significant difference in the distribution of the two data sets (*p* = 0.6515).

### 3.2. Accuracy of Inferring Genotypes

#### 3.2.1. A-SNP

When two-person mixtures were analyzed with bi-allelic SNPs, the five possible genotype combinations for each locus (i.e., identical homozygotes, a heterozygote of the minor contributor and a homozygote of the major contributor, a homozygote of the minor contributor and a heterozygote of the major contributor, different homozygotes and identical heterozygotes) yielded different theoretical *F_MAR_* values, as shown by the five lines in Figure 1. This was the kernel to design the strategy of deconvolution. However, there may be some special cases for the mixtures of close relatives. Parents and children share one allele per locus, and siblings may even share both alleles [42,43,44]. As a result, there are no different homozygotes between parents and children (i.e., only four possible combinations per locus in parent–child mixtures), and there may be few or even no different homozygotes between siblings in a limited number of SNP markers. Thus, the study also generated a number of in silico mixtures involving relatives. Among the six samples, P4 is the daughter of P1 and P2, and P2 and P3 are sisters. Some differences were found between parent–child mixtures and other mixtures in the results of deconvolution analysis, while no such differences were found for sister mixtures (two different homozygotes between P2 and P3). In the following, the results of non-parent–child mixtures were described in detail, and then those of parent–child mixtures (two at the ratio of 1:1, four at each of the other ratios) were presented.

As the mixture ratio changed, it was found that the points of scatterplots for *F_MAR_* values were clustered into three groups first (between 1:1 and 1:1.25), showed no obvious clustering trend (between 1:1.5 and 1:5), and finally were clustered into two groups (between 1:6 and 1:19). This was mainly caused by the variation rules of theoretical *F_MAR_* values at different ratios (i.e., the clustering trend of the five lines in Figure 1). However, the points in the scatterplots of the parent–child mixtures could be clustered into two groups at the ratio of 1:2.75.

**Figure 5:**
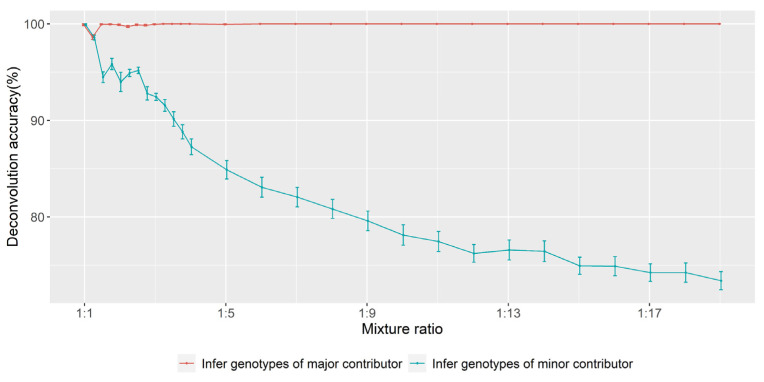
Deconvolution accuracy of non-parent–child in silico mixtures. The points represent the mean accuracy per ratio, and the error bars represent the standard error of the mean.

For the deconvolution of in silico mixtures, the accuracy of evenly balanced simulations was higher than that of unevenly balanced simulations, and the accuracy of inferring profiles for the major contributor was higher than that of inferring profiles for the minor contributor (Figure 5).

With regard to evenly balanced simulations, the deconvolution of 1:1 mixtures performed better than 1:1.25 mixtures. In all the 1:1 mixtures, only one heterozygote at rs2342747 was incorrectly inferred as a homozygote due to too low coverage (52×). When the proportion of two contributors changed slightly to 1:1.25, it caused a decrease of *TF* generated by the combination from the homozygote of the minor contributor and the heterozygote of the major contributor (i.e., the decline in *TF4* of Figure 1). In addition, compared with the case of the 1:1 ratio, the actual *F_MAR_* values of *TF4* could be further reduced due to the heterozygote imbalance at some loci, which was easier to be incorrectly clustered into the lower category and eventually resulted in more errors. For example, there were several errors at rs737681, and the *F_MAR_* values of heterozygote at this locus were higher than 60% in the original single samples. 

**Figure 6:**
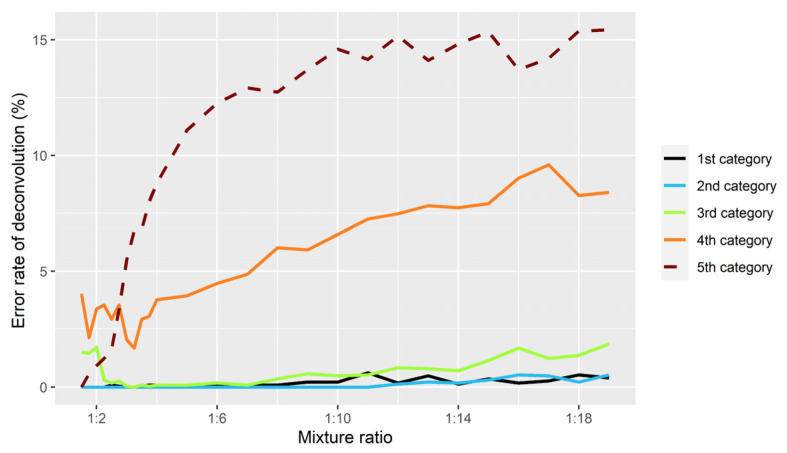
Error rate of KM5.R and KM2.R’s deconvolution analysis in each category for inferring the profiles of the minor contributors in non-parent–child in silico mixtures.

With regard to unevenly balanced simulations, the strategies used to infer the genotypes of the major contributor worked well. This study created a total of 780 non-parent–child mixtures at ratios between 1:1.5 and 1:19, where 763 mixtures were inferred correctly and 17 mixtures had only one error each. The errors were mainly concentrated in 3rd category that was clustered with KM5.R. This was because the actual *F_MAR_* values generated by the combinations of the same heterozygotes were higher than the theoretical values due to allelic imbalance. However, the performance of inferring genotypes of the minor contributor was different. As the proportion of the minor contributor became lower, it was more difficult to infer the genotypes. Figure 6 illustrates error rate of KM5.R and KM2.R’s deconvolution analysis in each category for inferring the profiles of the minor contributor. It can be seen that 1st, 2nd, and 3rd categories performed stably without too many mistakes; 4th and 5th categories made more mistakes, and the number of mistakes gradually increased with the reduction proportional of the minor components. There were two reasons for the excessive errors in 4th and 5th categories. On the one hand, *TF* from these two categories became closer in unevenly balanced mixtures (i.e., *TF4* and *TF5* in Figure 1). On the other hand, in addition to the original heterozygote imbalance at some loci, extracting sequencing reads for simulation exacerbated the imbalance. Therefore, the K-means clustering algorithm was prone to confuse some genotypes of 4th and 5th categories.

The deconvolution of evenly balanced parent–child mixtures and the inference of genotypes for the major contributors of the unevenly balanced parent–child mixtures performed well. However, the accuracy of inferring genotypes of the minor contributors was lower than other mixtures due to the lack of different homozygotes in the combination (see Supplementary File S1).

#### 3.2.2. Y-SNP

All Y-SNPs from both evenly balanced and unevenly balanced in silico mixtures were correctly inferred, regardless of major or minor contributors’ genotypes.

### 3.3. Estimated Ratio

**Figure 7:**
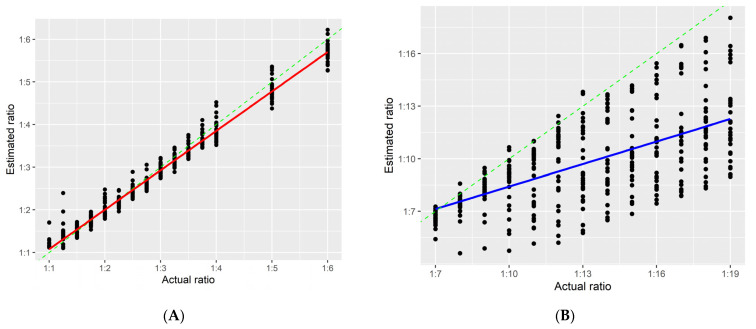
The actual ratios (*x*-axis) of 825 in silico mixtures against their estimated ratios (*y*-axis). (**A**,**B**) show mixture ratios of 1:1 to 1:6, and 1:7 to 1:19, respectively. The green dashed line represents the diagonal line of the scatterplots, and the red and blue solid lines are linear regression lines. The red line was intercepted on the *y*-axis at 0.1522, and the slope was 0.9251, with a correlation coefficient *R^2^* = 0.9798 (*p* < 0.05). The corresponding values for the blue line were 4.1315, 0.4282, and 0.3964 (*p* < 0.05), respectively.

Since the *F_MAR_* value from the different genotype combination of Y-SNPs in a two-person mixture was close to the proportion of the major contribution, using that to estimate mixture ratios is simple. However, this method fails if there are no men, or only one man, or two identical Y-SNPs in the mixture. Thus, the study used *F_MAR_* values of A-SNPs and Equation (2) to estimate mixture ratios. The reason for using *TF2* in Equation (2) to calculate ratios was that the central value of the 2nd category of KM3.R, KM5.R, and KM2.R was closer to the theoretical value, resulting in the fewest errors. The calculated results were almost equal to the actual ratios in the range of 1:1 to 1:6, but the method did not perform very well for the remaining ratios (Figure 7).

### 3.4. Validation

As shown in Table 3, the analysis results of the in vitro mixture were close to those of the in silico mixture in terms of deconvolution accuracy and estimated ratio. Moreover, all the Y-SNPs of the true mixture were deconvolved correctly.

### 3.5. Determination of Major and Minor Contributors

Before inferring the unknown profile, it is necessary to determine whether the known person is a major or minor contributor in an unevenly balanced mixture. This can be realized through our study by using the features of scatterplots, the clustering of known profiles, and the estimated mixture ratio, etc. The scatterplots of unevenly balanced mixtures were divided into the scenarios of no aggregation trend (Figure 2B) and aggregation to two categories (Figure 2C). If all points of a scatterplot of the *F_MAR_* values obviously concentrated in two regions, and the known genotypes of 1st, 2nd, and 3rd categories were all homozygotes, then the known person should be the major contributor. In another case, if all points had no obvious clustering trend, the known genotypes of the 2nd category are all heterozygotes, then the known person should be the minor contributor (see Figure 3). Most of the mixtures could be analyzed by the method described above. However, several special cases were found in deconvolution analysis for the mixtures at the ratio of 1:1.5, where both homozygotes and heterozygotes appeared in the 2nd category after the execution of the script KM5.R. When the proportions of major and minor contributors did not differ much, there was a small gap between *TF2* and *TF4*, which led to poorer clustering results. Fortunately, there was still a solution for this particular case. If there were more heterozygotes in the 2nd category and more homozygotes in the 3rd category, then the known genotypes can be determined to be from the minor contributor. In general, determining whether a known person is a major or minor contributor requires several logical judgments using all the information obtained.

## 4. Conclusions

The study described the scripts KM3.R, KM5.R, and KM2.R for deconvolving A-SNPs and estimating mixture ratios, and Y-KM2.R for deconvolving Y-SNPs. They showed that deconvolution was highly accurate for interpreting evenly balanced mixtures or unevenly balanced mixtures with the known profile from the minor contributor. The mixture ratios were also accurately estimated in the range of 1:1 to 1:6. The general workflow of mixture analysis is as follows. First, a scatterplot of *F_MAR_* is made after a two-person mixture profile is obtained. Based on the distribution of the plots, the K-means clustering method is used to cluster the *F_MAR_* values and estimate the mixture ratio. Then, it is determined whether the known profile is from the major or minor contributor, and the corresponding strategy is used to infer the genotype. Meanwhile, the accuracy of the inferred profiles is evaluated according to the mixture ratio. Next, the suspect is found by matching the inferred profile with the suspect profile or searching the database for similar profiles. Finally, LR is calculated to determine the weight of evidence according to the International Society of Forensic Genetics (ISFG) [15].

While there were considerable challenges for accurately inferring the minor contributor profile in a severely imbalanced mixture, the deconvolution analysis for that was still somewhat informative. Since the loci with deconvolution errors of the minor contributor mainly concentrated in the 4th and 5th categories, the others had relatively high accuracy, which may be useful for matching the suspect profile. There were two reasons for these errors: the low sequencing coverage and the imbalanced heterozygote coverage. Notably, low coverage can further aggravate the heterozygote imbalance. Buchard et al. suggested that the analysis criteria for minimum coverage was set at 200 reads in order to ensure small variations in the heterozygote balances [28]. The study also observed that the deconvolution of loci with a coverage of less than 200× was more prone to errors (e.g., rs2342747 mentioned in Section 3.2.1). It has been reported that both low DNA input (≤100 pg) and high PCR cycle number (>23) in library construction resulted in poor performance of the heterozygote balances [27,45]. Meiklejohn et al. mentioned that the homopolymer stretch immediately flanking the SNP position affected the heterozygote balances [46], which we also found at rs737681. It is well-known that the Ion Torrent system has a difficulty in homopolymer sequencing [17,47]. Therefore, to improve the accuracy of inferring minor contributor profile in mixed stain, the next study will try to find the optimal experimental conditions and find more SNPs with well-balanced heterozygote reads.

In addition, the method for generating in silico mixtures in our study performed well and is equally applicable to the study of other markers, such as microhaplotype and mitochondrial DNA that can be used to analyze mixtures of more than two people.

## Figures and Tables

**Table 1:** Mixing two random individuals at the ratio of 1:1.

Individual 1	Individual 2	Mixture Profile	M1	M2	*TF* (%)	Mixture Deconvolution
AA	AA	AAAA	A	-	100	identical Hom (M1/M1)
AA	AG	AAAG	A	G	75	Hom & Het (M1/M1 & M1/M2)
AA	GG	AAGG	A or G	G or A	50	different Hom (M1/M1 & M2/M2)
AG	AA	AAAG	A	G	75	Het & Hom (M1/M2 & M1/M1)
AG	AG	AAGG	A or G	G or A	50	identical Het (M1/M2)
AG	GG	AGGG	G	A	75	Het & Hom (M1/M2 & M1/M1)
GG	AA	AAGG	A or G	G or A	50	different Hom (M1/M1 & M2/M2)
GG	AG	AGGG	G	A	75	Hom & Het (M1/M1 & M1/M2)
GG	GG	GGGG	G	-	100	identical Hom (M1/M1)

Note: M1 denotes the allele with the maximum amount; M2 denotes the allele with the minimum amount; Hom & Het denotes that the locus is mixed by a homozygote and a heterozygote.

**Table 2:** Relationship among k_co_, *TF*, and the genotype combinations.

k_co_	*TF*	Minor & Major
*n* + 1	*TF1*	identical Hom
*n*	*TF2*	Het & Hom
*n* − 1	*TF3*	different Hom
1	*TF4*	Hom & Het
0	*TF5*	identical Het

**Table 3:** The deconvolution accuracy of A-SNPs in 9948 and the estimated mixture ratio.

2800 M and 9948	Accuracy (%)	Estimated Ratio
19:1	58.62	11.17:1
9:1	87.37	8.84:1
4:1	89.66	3.56:1
1:1	98.85	1:1.06
1:4	100	1:4.04
1:9	100	1:5.23
1:19	100	1:7.95

## Supplementary Materials

The following supporting information can be downloaded at: https://www.mdpi.com/article/10.3390/genes13050884/s1. File S1: Supplementary Figures; File S2: Details of 825 in silico mixtures. File S3: Pipeline for simulation. File S4: Python script genoinfo.py.
