# Supplementary material for: A New Computational Deconvolution Algorithm for the Analysis of Forensic DNA Mixtures with SNP Markers
Source: Genes (Basel). 2022 May 15;13(5):884. doi: 10.3390/genes13050884 (PMC9141285; doi:10.3390/genes13050884)
Supplement: Supplementary file 1 [file genes-13-00884-s001.zip › File_S1_Figures.pdf]

Supplementary Figures

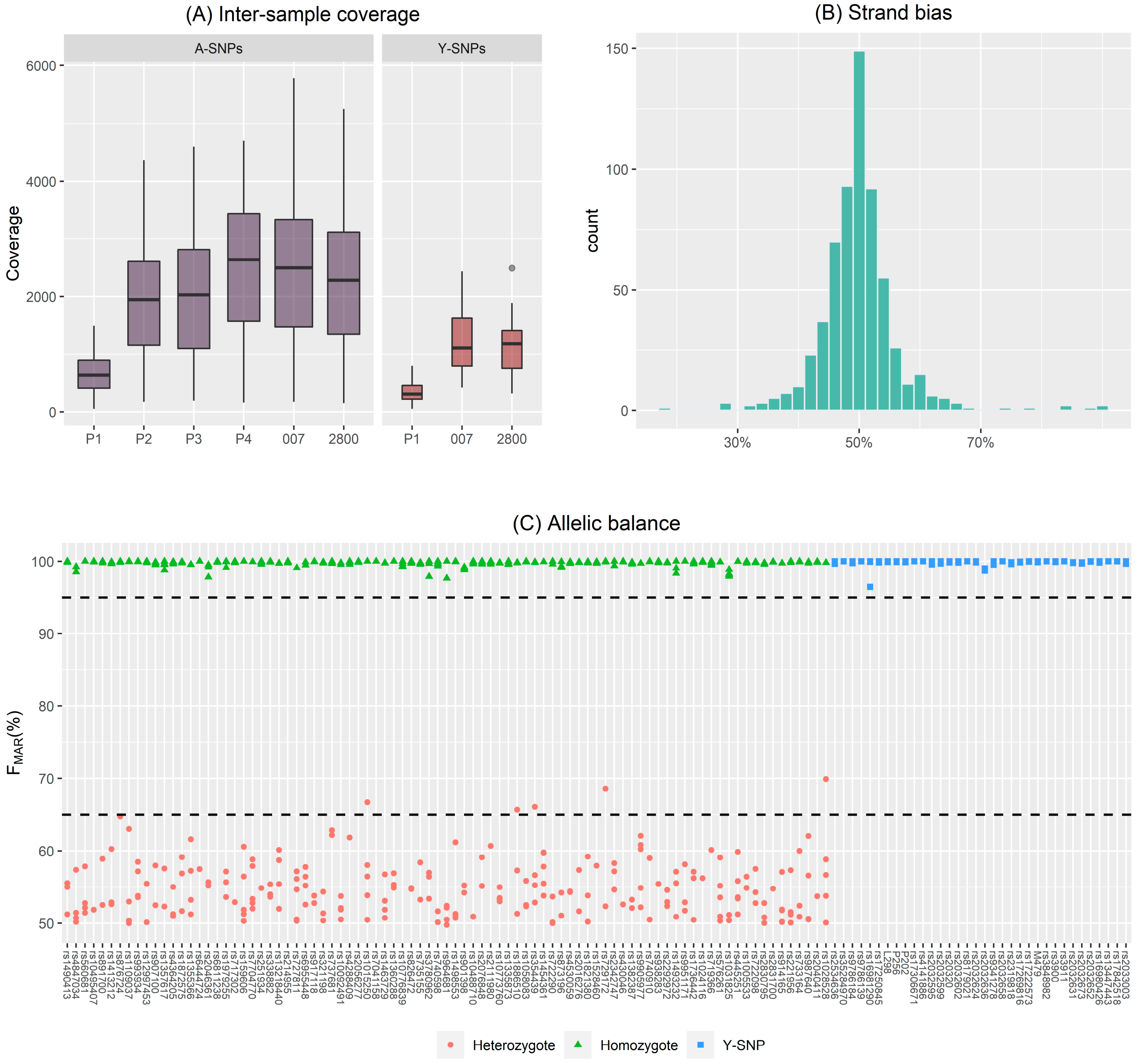

Figure S1. The overall performance of the six single-source samples in terms of coverage, strand bias, and FMAR. (A) The Box plots show the distribution of the inter-sample coverage of A-SNPs and Y-SNPs. The coverage of P1 had the smallest mean and standard deviation (A-SNPs: 675×±328×, Y-SNPs: 351×±173×). (B) The histogram illustrates the performance of strand bias for the six samples. Eleven of the total 624 values (i.e., 1.76% of all values) were outside the threshold between 30% and 70%. (C) The scatter plot indicates the allelic balance of the six samples. The x-axis represents 121 loci, the y-axis represents the FMAR values, and the dashed lines at 95% and 65% represent the threshold for homozygotes and heterozygotes, respectively. Five of the total 232 heterozygotes exceeded the threshold, accounting for 2.16%.

Basic strategy of Y-KM2.R

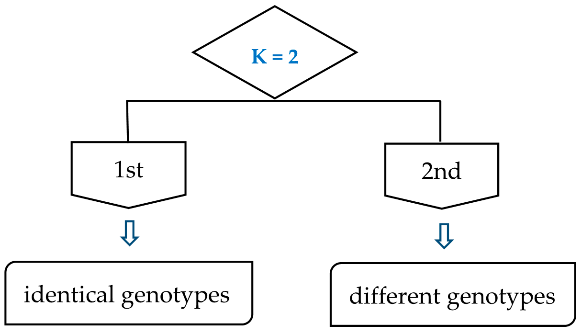

Figure S2. Flow chart of the basic strategy utilized for Y-SNPs deconvolution with the K-means clustering method. The value of K indicates the number of clusters needed for the dataset consisting of the F_{MAR} values of different loci of Y chromosome. The locus in the 1st category that had the higher central value was mixed by the identical genotypes, the locus in the 2nd category that had the lower central value was mixed by different genotypes. The central value of the 2nd category of Y-SNPs was close to the proportion of the major contribution in a mixture.

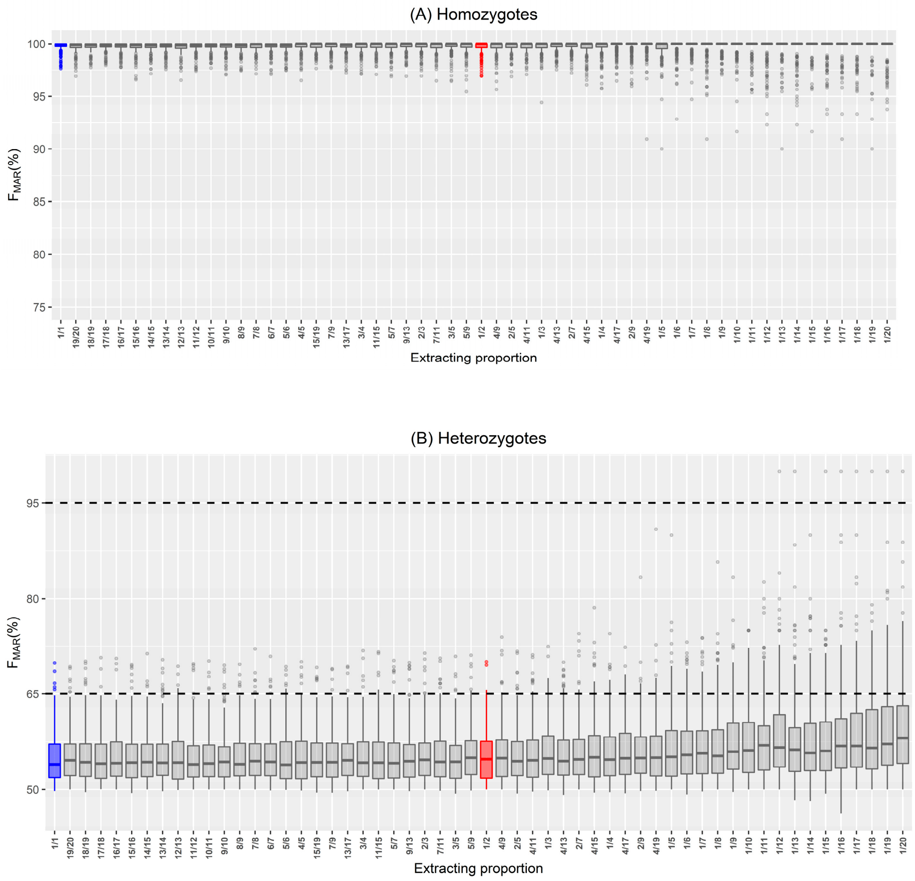

Figure S3. Distributions of the F_{MAR} values of homozygotes and heterozygotes in proportion files. The blue boxes represent the values of six original sample files, and the red boxes represent the values of 1/2 proportion files.

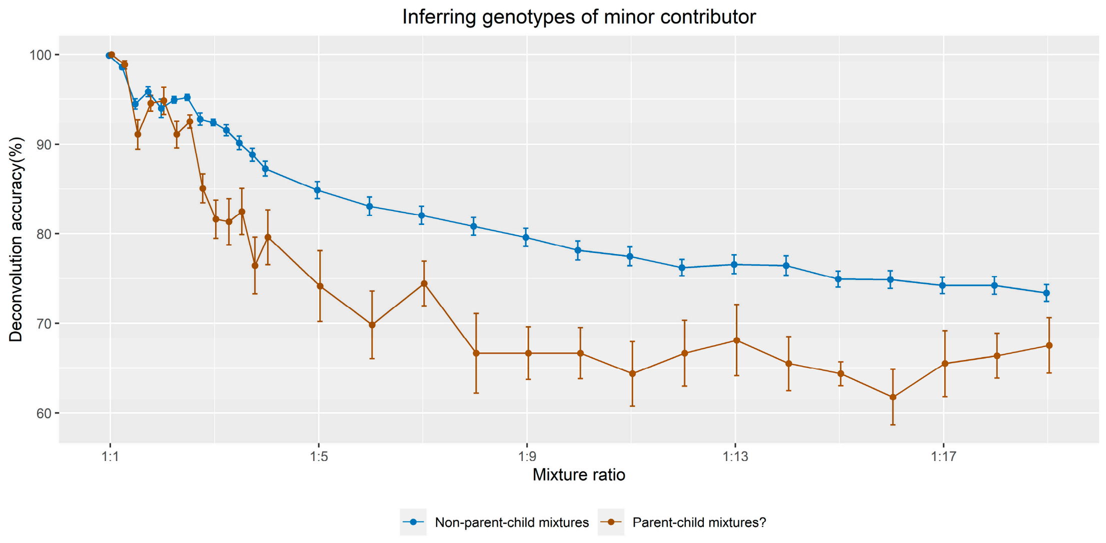

Figure S4. Comparison of the deconvolution accuracy of non-parent-child in silico mixtures with that of parent-child in silico mixtures in inferring the genotypes of the minor contributors. The points represent the mean accuracy of each mixture ratio, and the error bars represent the standard error of the mean.
