# Supplementary material for: A New Computational Deconvolution Algorithm for the Analysis of Forensic DNA Mixtures with SNP Markers
Source: Genes (Basel). 2022 May 15;13(5):884. doi: 10.3390/genes13050884 (PMC9141285; doi:10.3390/genes13050884)
Supplement: Supplementary file 1 [file genes-13-00884-s001.zip › File_S3_PipelineForSimulation.pdf]

Pipeline For Simulation

The command line started with “$” and was followed by the corresponding explanation starting with “#”. All command lines are run on the Linux operating system.

1. Several Important Command Lines of Splitting BAM Files

$ samtools view P1.bam|cut -f3,4|uniq -c |awk -F “ ” “$1>5{print$0}” > count_5.txt

The “view” command of SAMtools was used to view the P1.bam file, and the Linux command “cut” together with argument “-f” was applied to extract the position information of each sequence (i.e., two columns RNAME and POS). Then “uniq” with “-c” counted the number of occurrences of the same leftmost mapping position, “awk” with “-F” selected the position with more than 5 occurrences. Finally these sequence positions were stored in count_5.txt. Therefore, The target regions for amplification of the 121 loci could be found in count_5.txt.

$ samtools view P1.bam chr1:4367256-4367323 > subfile_first.sam

The target region for amplification of rs490413 located at chr1:4367256-4367323, and all sequences in this region were copied to subfile_first.sam using the command “samtools view”. Then, other sub-files were created in the same way.

2. Several Important Command Lines of Extracting and Merging

The reads of rs490413 and the reads of rs4847034 were stored in subfile_first.sam and subfile_second.sam alone, and their coverage in the P1 sample was 554× and 471×, respectively. Firstly, randomly extracting 277 sequencing reads (half of 554) from subfile_first.sam, and randomly extracting 236 reads (approximately half of 471) from subfile_second.sam, separately stored in half_first.sam and half_second.sam. Then, the rest of the sub-files were processed in the same way. After a series of commands processing, the 121 newly generated files were combined into a new BAM file named P1_half.bam. Therefore, the coverage of each locus in P1_half.bam was half of that of P1.bam. In addition, P2_half.bam, P3_half.bam, P4_half.bam, 007_half.bam, 2800M_half.bam were produced in the same way, and the coverage per locus of these files was the same as that of P1_half.bam. Finally, in silico mixture of P1 and P2 at 1:1 ratio, was created by merging P1_half.bam and P2_half.bam. The above workflow was utilized to generate all the in silico mixtures.

$ shuf subfile_first.sam -n 277 -o half_first.sam

The Linux command “shuf” randomly extracted 277 sequencing reads from subfile_first.sam, storing them in half_first.sam.

$ shuf subfile_second.sam -n 236 -o half_second.sam

The Linux command “shuf” randomly extracted 236 sequencing reads from subfile_second.sam, storing them in half_second.sam.

$ samtools view -H P1.bam > P1_header.sam

Output the header section of P1.bam to P1_header.sam.

$ cat P1_header.sam half_* > P1_haft.sam

Combined P1_header.sam and all extracted files into P1_haft.sam.

$ samtools view -bS P1_half.sam | samtools sort -o P1_half.bam

After P1_half.sam had been converted and sorted, P1_half.bam was created. P2_half.bam was created in the same way, and the coverage per locus of this file was the same as that of P1_half.bam.

$ samtools merge -cp P1_P2_1_1.bam P1_half.bam P2_half.bam

In silico mixtrue of P1 and P2 at 1:1 ratio , named P1_P2_1_1.bam, was created by merging P1_half.bam and P2_half.bam.

$ samtools sort P1_P2_1_1.bam -o P1_P2_1_1.bam

Sorted P1_P2_1_1.bam.
